# Digestive Enzyme Activities and Gut Emptying Are Correlated with the Reciprocal Regulation of TRPA1 Ion Channel and Serotonin in the Gut of the Sea Urchin *Strongylocentrotus intermedius*

**DOI:** 10.3390/biology11040503

**Published:** 2022-03-24

**Authors:** Jingyun Ding, Huiyan Wang, Zequn Li, Jiangnan Sun, Peng Ding, Xiaomei Chi, Mingfang Yang, Yaqing Chang, Chong Zhao

**Affiliations:** 1 Key Laboratory of Mariculture & Stock Enhancement in North China’s Sea, Ministry of Agriculture and Rural Affairs, Dalian Ocean University, Dalian 116023, China; jingyunding111@outlook.com (J.D.); wanghuiyan3@gmail.com (H.W.); lzq678673138@gmail.com (Z.L.); jnsun77@gmail.com (J.S.); 1712913708dp@gmail.com (P.D.); xiaomeichi0815@outlook.com (X.C.); mingfangyang0307@outlook.com (M.Y.); 2 Southern Marine Science and Engineering Guangdong Laboratory, Guangzhou 511458, China

**Keywords:** sea urchin, TRPA1, 5-HT, digestive enzyme, gut emptying

## Abstract

**Simple Summary:**

Sea urchins are key in the trophic cascades of benthic communities. They also possess physiological characteristics of low digestibility. Thus, we explored the relationship between the indicators (digestive enzymes activities and the gut emptying) and molecules (TRPA1 and 5-HT) related to digestive physiology in the sea urchin *Strongylocentrotus intermedius*. The results in the present study indicate that digestive enzyme activities and gut emptying are correlated with the TRPA1 and 5-HT in the gut of *S. intermedius.* This novel finding not only is important for understanding the low food digestibility of sea urchins but provides an entry point to further investigate the molecular details of digestive physiology of sea urchins.

**Abstract:**

The energetic link in the benthic community is based on physiological characteristics of the low food absorption efficiency of sea urchins. Low food absorption efficiency of sea urchins is correlated with the activity of digestive enzymes and the duration of food in their gut. Thus, the digestive enzymes activities (pepsin and amylase enzyme activities) and gut emptying are important indicators in assessing nutrient digestion and absorption in sea urchins. In the present study, the relationship between these indicators and molecules related to digestive physiology were quantified in sea urchins. We found (1) an inter-regulatory relationship existed between Transient receptor potential cation channel, subfamily A, member 1 (TRPA1), and serotonin (5-hydroxytryptamine; 5-HT) in the gut of *Strongylocentrotus intermedius*; (2) digestive enzyme activities were negatively correlated with the TRPA1 and concentration of 5-HT in the gut of *S. intermedius*; (3) gut emptying rate was positively correlated with TRPA1 and concentration of 5-HT in the gut of *S. intermedius*. The present study revealed that the digestion and absorption of food are correlated with the TRPA1 and 5-HT in the gut of *S. intermedius*, which provides valuable information about the digestive physiology of sea urchins. This novel finding is relevant to understanding the low food digestibility of sea urchins. It also provides valuable information to the digestive physiology of sea urchins, which are key to maintaining the stability of food webs in the marine ecosystem.

## 1. Introduction

Sea urchins are major consumers of macroalgae in shallow waters [1], and play a key trophic role by capturing kelp and making it available to a suite of benthic detritivores [2]. They thus play an important role in regulating kelp forests ecosystem [3]. The low food digestibility (absorption efficiency) of sea urchins [4,5,6] affects their ecological function. Low food digestibility of sea urchins requires large food consumption for the survival and reproduction [7]. Due to the inefficient digestive system of sea urchins [8,9], a substantial portion of urchin feces is relatively unprocessed vegetative materials [10,11]. The feces are important sources of calories and enriched nutrients for nearby consumers [12,13]. Thus, sea urchin feces represent an important energetic in benthic community [9] and play a positive nutritional role in kelp ecosystem [2].

Low food digestibility of sea urchins is correlated with the activity of digestive enzymes [7] and the duration during which the food stays in their gut [14]. Digestive enzymes directly affect the absorption and utilization of nutrients in the sea urchin *Strongylocentrotus intermedius*, thereby affecting their growth [15,16]. Amylase and pepsin have been extensively studied as representative digestive enzymes of sea urchins and their activities have been used to assess the digestive and absorptive capacities of sea urchins [15,16]. Gastric emptying is a common method used to quantify gastric motility [17], which greatly affects the rate of digestion and absorption of nutrients [18,19,20,21]. Antarctic echinoderms have low-energy diets and require long periods of digestion [7]. They feed slowly to ensure enough gut-passage time for adequate enzymatic breakdown of food, thus providing more energy [7]. Therefore, digestive enzymes activities and gut emptying are appropriate indicators of digestion and absorption capacities of *S. intermedius*. However, the relationship between these indicators and molecules related to digestive physiology remains largely unknown in sea urchins.

Transient receptor potential cation channel, subfamily A, member 1 (TRPA1), a member of the TRP family, is expressed in sensory neurons and associated with somatosensation [22,23,24]. TRPA1 is abundantly expressed in the gastrointestinal tract of mammals and plays an important role as a molecular receptor in regulating their gastrointestinal functions [25,26]. *TRPA1* was also reported to be expressed in the gut of *S. intermedius* [27]. This suggests that TRPA1 is probably involved in regulating gut function in *S. intermedius*.

Serotonin (5-hydroxytryptamine; 5-HT) is an important signaling molecule that regulates animal gastrointestinal functions, including digestion and absorption [28,29]. The release of 5-HT coordinates the function of the gastrointestinal tract, which promotes digestion and absorption of nutrients in mammals [28], insects [30], and crustaceans [29]. Further, TRPA1 mediates the release of 5-HT to regulate gastrointestinal functions in rats, including gastric emptying and gastric motility [25,31]. Although the serotonergic nervous system was also reported in sea urchins [32], to our knowledge, 5-HT has not been investigated in digestive physiology of sea urchins. We hypothesized that a correlation exists between the indicators (digestive enzymes activities and the gut emptying) and molecules (TRPA1 and 5-HT) related to digestive physiology in sea urchins.

To test this hypothesis, we ask: (1) whether an inter-regulatory relationship exists between TRPA1 and 5-HT in the gut of *S. intermedius;* (2) whether a correlation exists between digestive enzyme activities and the reciprocal regulation of TRPA1 and 5-HT in the gut of *S. intermedius*; and (3) whether a correlation exists between gut emptying and reciprocal regulation of TRPA1 and 5-HT in the gut of *S. intermedius*.

## 2. Materials and Methods

### 2.1. Sea Urchins

Cultural conditions before the experiments: Sea urchins (test diameter: ~35 mm) were obtained from a local aqua-farm and transported to the Key Laboratory of Mariculture and Stock Affairs at Dalian Ocean University (38°52′ N, 121°34′ E). They were maintained at 13–14 °C in the laboratory in tanks (1000 L) at ambient temperature with sand filtered sea water. The temperature of the seawater in the laboratory corresponds to the temperature of the local aqua-farm where the sea urchins were collected. Sea urchins were fed freshly-collected *Saccharina japonica* and *Ulva lactuca ad libitum* before the experiments. One third of the seawater was changed every three days, with uneaten food and feces removed.

**Figure 1:**
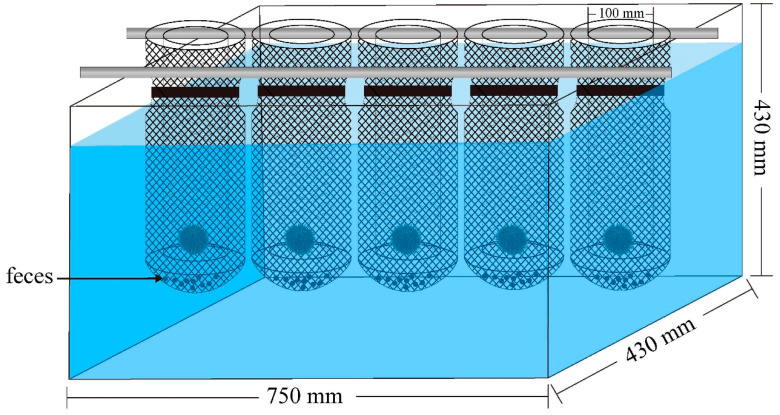
Diagram of the feces collection in each temperature-controlled tank.

Experimental culture conditions: *S. intermedius* (33.22 ± 0.06 mm test diameter; 13.63 ± 0.07 g body weight) were used for experiments 1–4 (more details see the Results section). For experiments 1, 30 individuals were randomly selected from the laboratory tank (1000 L) under the natural culture condition. For experiments 2–4, *S. intermedius* were randomly selected from the laboratory tank and placed in temperature-controlled tanks (750 × 430 × 430 mm, ~139 L; Huixin Co., Dalian, China). Five tanks were used for each treatment group, and five individuals were placed into an individual cylindrical plastic cage (diameter: 100 mm, mesh size: 8 mm) in each tank (*n* = 5). Thus, 25 temperature-controlled tanks are in total for experiment 2, and 35 temperature-controlled tanks in total for experiment 3 and 4, respectively. Sea urchins used in experiments 2–3 were fed with fresh macroalgae *Undaria pinnatifida*. Sea urchins for experiment 4 were not fed for two weeks until the experiments started. We attached a net bag to the outside of each cage to collect the 48-h accumulative feces (Figure 1). The plastic cage and its outer net bag allowed free water exchange in each tank. Experiments 2–4 were carried out under illumination of ~300 lx and at the seawater temperature of 14.8–15.1 °C. The natural photoperiod was 10 h light: 14 h dark.

### 2.2. Chemicals

**Figure 2:**
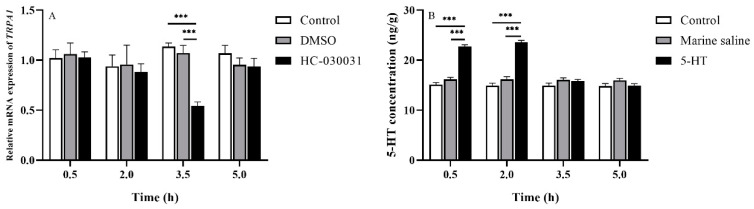
(**A**) *TRPA1* expression in the gut of *Strongylocentrotus intermedius* after the HC-030031 injection (*n =* 5). (**B**) The 5-HT level in the gut of *S. intermedius* after the 5-HT injection. The symbol *** means *p <* 0.001 (*n =* 5, mean ± s.e.m.).

HC-030031, a TRPA1 selective antagonist, inhibited TRPA1 activity in vivo and in vitro [33] (Aladdin, Shanghai, China), dimethyl sulfoxide (DMSO) (Aladdin, Shanghai, China), stroke-physiological saline solution for marine animals (2% sodium chloride solution; Leagene, Beijing, China), and 5-HT (Sigma-Aldrich, Shanghai, China), were used in this study. HC-030031 and 5-HT were dissolved in 10% DMSO in distilled water and stroke-physiological saline solution for marine animals (marine saline), respectively. The dosage of chemicals in experiments 2, 3 and 4 were as follows: HC-030031 (2.5 mg/mL, 40 μL/ind); 10% DMSO (40 μL/ind); 5-HT (25 μg/g ind^−1^, the solution volume depended on the body weight of different individuals) [29]; marine saline (the same solution volume as 5-HT/ind). According to our preliminary experiment, the most effective drug durations were 2–3.5 h (*df*1 = 2, *df*2 *=* 12, *F* = 35.518, *p* < 0.001, Figure 2A) for HC-030031 and 0–0.5 h for 5-HT (*df*1 = 2, *df*2 = 12, *F* = 129.133, *p* < 0.001, Figure 2B) after injection for HC-030031 and 5-HT, respectively. For experiments 2, 3 and 4, all chemicals were injected into the peristomial membrane of *S. intermedius* [34] by using micro sampling syringes. To avoid cross-contamination, each chemical corresponded to its own individual syringe.

### 2.3. Sample Collection for TRPA1 Expression, 5-HT Concentration and Enzyme Activities

For each experiment, we carefully collected gut samples at the same time. For experiment 1, each sea urchin’s gut tissue was divided into three samples. The three samples were stored in separate sterile microcentrifuge tubes for the subsequent analysis of *TRPA1* expression, 5-HT concentration, and digestive enzyme activities, respectively. For experiment 2, gut tissues were collected from each *S. intermedius* with the HC-030031 injection for the subsequent analysis of 5-HT concentration. The gut tissues from each individual with the 5-HT injection were used for the subsequent analysis of *TRPA1* expression. For experiment 3, the gut sample of each sea urchin was used for the subsequent analysis of digestive enzyme activities.

The samples for transcriptional analysis of *TRPA1* expression and digestive enzyme activities were immediately snap-frozen in liquid nitrogen, and then stored at −80 °C until the extraction of RNA and determination of digestive enzyme activities. The samples for the analysis of 5-HT concentration were immersed in the 0.01 M phosphate buffer solution (PBS, pH = 7.4) and then stored at −20 °C until the determination of 5-HT concentration.

### 2.4. Total RNA Extraction and cDNA Synthesis

Total RNA was isolated from all samples obtained in the above experiments according to the instruction of RNAprep pure Tissue Kit (Tiangen, Beijing, China). The integrity of RNA was shown by visualization on agarose gel, whose concentration and quantity were evaluated by spectrophotometer (NanoPhotometer, Munich, Germany). The cDNA was synthesized using the PrimeScript™ RT reagent Kit (TaKaRa, Dalian, China) in a 20 μL reaction system with 1000 ng total RNA, 4 μL 5 × PrimeScript™ buffer, 2.5 pmol/μL Oligo dT Primer, 5 pmol/μL Random 6 mers and 1 μL PrimeScript RT Enzyme Mix I. Reactions were incubated at 37 °C for 15 min, followed by a final 5 s denaturation at 85 °C to deactivate the enzyme. All cDNA samples were stored at −20 °C for quantitative real-time PCR.

### 2.5. Transcriptional Analysis of TRPA1

The expression of *TRPA1* in all experiments was analyzed by quantitative real-time PCR (qRT-PCR), which performed in triplicate using the Applied Biosystem 7500 Real-time system (Applied Biosystem, Foster, CA, USA). According to the manufacturer’s instructions, the reaction volume was 20 μL containing 2 μL of 1:5 dilution cDNA, 6 μL Nuclease-free Water, 10 μL TB Green Premix Ex Taq II, 0.4 μL of ROX Reference Dye II (TB Green™ Premix Ex Taq™ II; TaKaRa, Dalian, China), 0.4 μM of each primer (Table 1). The qRT-PCR program included 95 °C for 30 s, followed by 40 cycles of 95 °C for 5 s, and 60 °C for 32 s. Amplification products were analyzed by melting curve at the end of each PCR to confirm amplification specificity. In this study, 18S rRNA gene was used as the reference gene [34,35]. The relative expression level of *TRPA1* was calculated using the comparative Ct method (2^−^^△△CT^ method) [36].

### 2.6. Analysis of 5-HT Concentration

The gut samples obtained in the above experiments were weighed and then minced to small pieces which were homogenized in 0.01 M phosphate buffer solution (PBS, pH = 7.4) with a glass homogenizer on ice. The volume depended on the weight of the tissue. Nine mL PBS was added in the 1 g gut sample. The homogenates were then centrifuged for 5 min at 5000× *g* to acquire the supernatant. The supernatant was used for further analysis of 5-HT concentration. The 5-HT concentration was measured by using the 5-Hydroxytryptamine (5-HT) ELISA kit (Mlbio, Shanghai, China). The determination principle, method and unit definition of 5-HT are in accordance with the instructions of the kit. The absorbance of samples at 450 nm was measured using the SpectraMax i3x (Molecular devices, Wals, Austria).

### 2.7. Analysis of Digestive Enzyme Activities

The samples of the gut obtained in the above experiments were immediately added to a precooled glass blender in an ice bath, after which physiological saline solution (0.85% sodium chloride) was added. The addition ratio is 1 g of sample to 10 mL of 0.85% sodium chloride solution [37]. The homogenate was centrifuged at 4500× *g* for 40 min at 4 °C. The supernatant was used for the analysis of digestive enzyme activities. The activities of enzymes were measured using the kits of amylase and pepsin (Nanjing Jiancheng Bioengineering Institute, Nanjing, China). The determination principle, method and unit definition of pepsin and amylase activities refer to the kit instructions. The samples were measured by a visible spectrophotometer (VIS7200A; Shanghai Techcomp Instrument Ltd., Shanghai, China) at the absorbance of 600 nm for determination of amylase activity and 680 nm for determination of pepsin activity.

### 2.8. Statistical Analysis

All data were tested for normal distribution and homogeneity of variance using Kolmogorov-Smirnov test and Levene test, respectively. Data from experiment 1 were analyzed using independent sampled t-test. Data from experiments 2, 3 and 4 were analyzed using one-way ANOVA. Fisher’s least significant difference (LSD) multiple comparisons were conducted to compare significant differences among treatments when a significant effect was found. All data are expressed as mean values ± standard error (mean ± s.e.m.). All statistical analysis was performed using SPSS 22.0 statistical software. The level of significance was considered as *p* < 0.05.

## 3. Results

### 3.1. Experiment 1: TRPA1 Is Involved in the Food Digestion of S. intermedius

**Figure 3:**
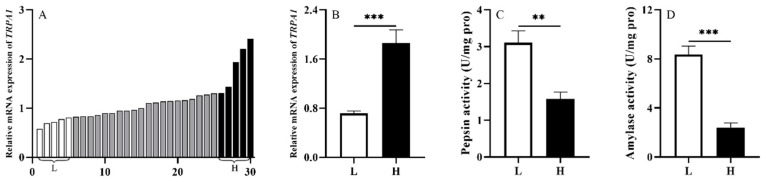
TRPA1 is involved in food digestion of *Strongylocentrotus intermedius*. (**A**) The expression of *TRPA1* in the gut of *S. intermedius* (*n =* 30). (**B**) *TRPA1* expression of groups L and H (*n =* 5). (**C**,**D**) The activities of pepsin and amylase in the gut of the groups L and H (*n =* 5). The symbols ** and *** mean *p <* 0.01 and *p <* 0.001, respectively (mean ± s.e.m.).

To investigate whether TRPA1 is involved in the food digestion of *S. intermedius*, we randomly selected 30 sea urchins from the laboratory tank (1000 L) under the natural culture condition and measured *TRPA1* expression level of their guts. Subsequently, we ranked the expression levels of *TRPA1* of the 30 sea urchins, and compared the 5 sea urchins with the highest expression levels of *TRPA1* (named as group H) with the 5 with the lowest expression levels of *TRPA1* (named as group L). Then, we measured the pepsin and amylase enzyme activities of intestinal samples with the corresponding groups H and L. The expression of *TRPA1* in the guts of 30 sea urchins was ranked from low to high (from left to right) (*n* = 30, Figure 3A).

We found that sea urchins of group H exhibited significantly higher expression of *TRPA1* than those of group L (*df* = 8, *t* = 5.258, *p* < 0.01, Figure 3B). The pepsin and amylase activities of sea urchins in group L were significantly higher than those in group H (*df* = 8, *t* = −4.140, *p <* 0.01; *df* = 8, *t* = −7.745, *p <* 0.001) (Figure 3C,D). These results showed that TRPA1 is involved in the food digestion of *S. intermedius*.

### 3.2. Experiment 2: An Interregulatory Relationship Exists between TRPA1 and 5-HT in the Gut of S. intermedius

**Figure 4:**
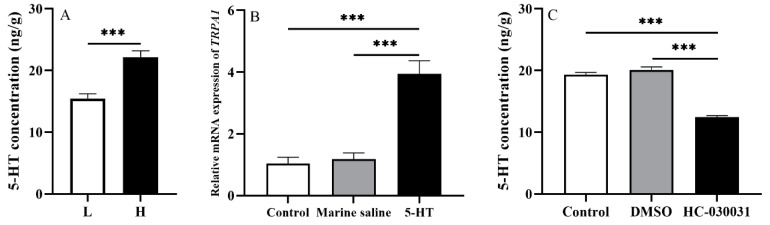
An inter-regulatory relationship between TRPA1 and 5-HT in the gut of *Strongylocentrotus intermedius*. (**A**) The 5-HT level in the gut of groups L and H (*n =* 5). (**B**) *TRPA1* expression after the 5-HT injection (*n =* 5). (**C**) The 5-HT level after the HC-030031 injection (*n =* 5). The symbol *** means *p <* 0.001, (mean ± s.e.m.).

The 5-HT concentration of groups H and L was measured accordingly in experiment 1 (*n =* 5). We found that 5-HT concentration of group H was significantly higher than that of group L (*df* = 8, *t* = 5.336, *p <* 0.001, Figure 4A).

To further investigate whether an inter-regulatory mechanism exists in the gut of *S. intermedius,* we firstly analyzed the 5-HT concentration in the guts of sea urchins injected with HC-030031. The average 5-HT concentration of the 5 individuals was considered as the 5-HT concentration for each temperature-controlled tank (*n =* 5). Five urchins in each of the 5 tanks were injected with 10% DMSO as the corresponding vehicle control group. Untreated sea urchins (without any chemical administration) in each of 5 tanks were used as the control group (*n =* 5). The results showed that *TRPA1* expression of sea urchins injected with 5-HT was significantly higher than those in the untreated ones and the ones injected with marine saline (*df*1 = 2, *df*2 = 12, *F* = 31.304, both *p <* 0.001, Figure 4B). No significant difference was found between the sea urchins injected with marine saline and the untreated sea urchins (*p >* 0.05).

We further analyzed the expression pattern of *TRPA1* in the guts of those injected with 5-HT. The average expression of *TRPA1* of the 5 individuals was considered as the *TRPA1* expression for each temperature-controlled tank (*n =* 5). Sea urchins were injected with marine saline as the corresponding vehicle control group (*n* = 5, named as the marine saline group). Untreated sea urchins (without any chemical administration) were used as the control group (*n* = 5). Sea urchins injected with HC-030031 exhibited significantly lower 5-HT concentration than those injected with DMSO and the untreated sea urchins (*df*1 = 2, *df*2 = 12, *F* = 112.016, both *p <* 0.001, Figure 4C). However, no significant difference was shown between sea urchins injected with DMSO and the untreated sea urchins (*p >* 0.05).

From the results in this experiment, we found that an interregulatory relationship existed between TRPA1 and 5-HT in the gut of *S. intermedius.*

### 3.3. Experiment 3: Digestive Enzyme Activities Are Negatively Correlated with TRPA1 and Concentration of 5-HT in the Gut of S. intermedius

Amylase and pepsin as representative digestive enzymes of sea urchins and their activities have been extensively used to evaluate the food digestion and absorption of sea urchins [15,16]. To investigate whether a correlation exists between digestive enzyme (amylase and pepsin) activities and the reciprocal regulation of TRPA1 and 5-HT in the gut of *S. intermedius*, we conducted a set of cascading experiments. The replicate set-up schemes of sea urchins were the same among all treatment groups in this experiment: 5 sea urchins in each of the 5 tanks. The average digestive enzyme activities of the 5 individuals were considered as the digestive enzyme activities for each temperature-controlled tank (*n =* 5).

**Figure 5:**
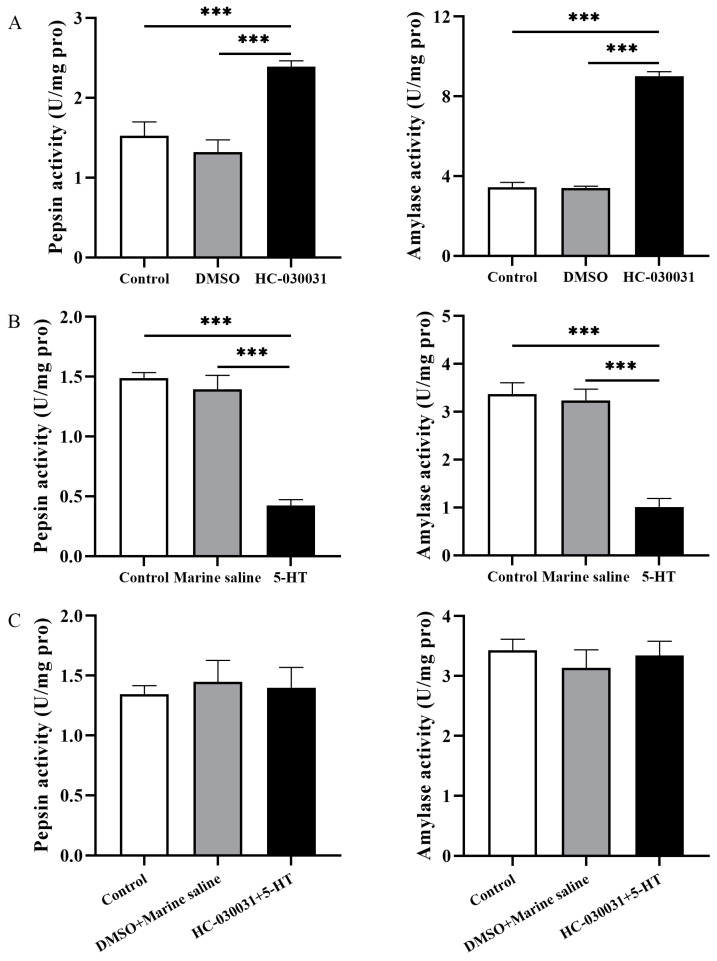
Digestive enzyme activities are negatively correlated with the reciprocal regulation of TRPA1 and 5-HT in the gut of *Strongylocentrotus intermedius.* (**A**) The activities of pepsin and amylase of the control, DMSO, and HC-030031 groups (*n =* 5). (**B**) The activities of pepsin and amylase of the control, marine saline, and 5-HT groups (*n =* 5). (**C**) The activities of pepsin and amylase of control, DMSO + marine saline, and HC-030031 + 5-HT groups (*n =* 5). The symbol *** means *p <* 0.001 (mean ± s.e.m.).

First, sea urchins were injected with HC-030031 to inhibit TRPA1 (named as the HC-030031 group). Sea urchins were injected with 10% DMSO as the corresponding vehicle control group (named as the DMSO group). Pepsin and amylase enzyme activities were individually measured for each group 3.5 h after the drug injection. Untreated sea urchins (without any chemical administration) were used as the control group. The HC-030031 group showed significantly higher pepsin and amylase activities than the DMSO and control groups (pepsin: *df*1 = 2, *df*2 = 12, *F* = 16.300, both *p <* 0.001; amylase: *df*1 = 2, *df*2 = 12, *F* = 274.045, both *p <* 0.001, Figure 5A). The activities were not significantly different between the DMSO and the control groups (both *p >* 0.05).

We then measured pepsin and amylase enzyme activities of the sea urchins 0.5 h after the 5-HT injection (named as 5-HT group). Sea urchins were injected with marine saline as the corresponding vehicle control group (named as the marine saline group). Untreated sea urchins (without any chemical administration) were used as the control group. The pepsin and amylase activities of 5-HT group were significantly lower than the control and marine saline groups (pepsin: *df*1 = 2, *df*2 = 12, *F* = 57.499, both *p <* 0.001; amylase: *df*1 = 2, *df*2 = 12, *F* = 36.704, both *p <* 0.001, Figure 5B). No significant difference was found between the control and marine saline groups (both *p >* 0.05).

Finally, sea urchins were injected with HC-030031, and then injected with 5-HT 3 h after the HC-030031 injection. After 3.5 h of the HC-030031 injection and 0.5 h of the 5-HT injection, sea urchins of the experimental group (named as HC-030031 + 5-HT group) were individually measured for their pepsin and amylase enzyme activities. DMSO + marine saline group was established as the vehicle control group accordingly. Untreated sea urchins (without any chemical administration) were used as control group. There were no significant difference of pepsin and amylase activities among the control, DMSO + marine saline, and HC-030031 + 5-HT groups (pepsin: *df*1 = 2, *df*2 = 12, *F* = 0.118, both *p >* 0.05; amylase: *df*1 = 2, *df*2 = 12, *F* = 0.364, both *p >* 0.05, Figure 5C).

These results show that digestive enzyme activities are negatively correlated with TRPA1 and concentration of 5-HT in the gut of *S. intermedius*.

### 3.4. Experiment 4: Gut Emptying Is Positively Correlated with TRPA1 and Concentration of 5-HT in the Gut of S. intermedius

Considering that gut emptying is another important indicator for food digestion and absorption of sea urchins, we further investigated whether a correlation exists between gut emptying and the reciprocal regulation of TRPA1 and 5-HT in the gut of *S. intermedius*. At the beginning of the experiment, 1.5 g of *U. pinnatifida* was fed to each sea urchin among different treatment groups [38] to ensure that they ate all the provided *U. pinnatifida* within 7 h and that they did not produce feces. The replicate set-up schemes of sea urchins and subsequent injection protocols were same as in the experiment 3. Finally, we removed the net bags containing the feces at the end of the 48-h experiment and put them in the oven at 110 °C (5 h) for drying to constant weight. The dry weight of the feces was measured by an analytical balance (AL204, Mettler-Toledo Instruments Co., Shanghai, China).

**Figure 6:**
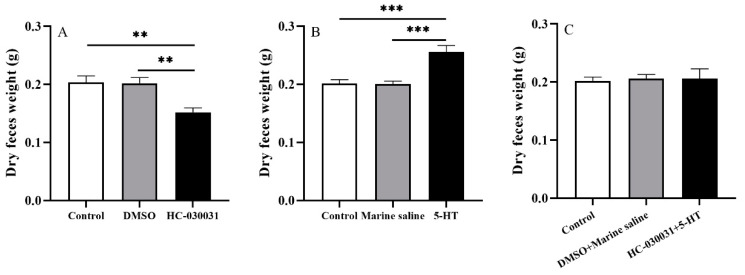
Gut emptying is positively correlated with the reciprocal regulation of TRPA1 and 5-HT in the gut of *Strongylocentrotus intermedius.* (**A**) Dry weight of the 48-h accumulated feces of the control, DMSO, and HC-030031 groups (*n =* 5). (**B**) Dry weight of the 48-h accumulated feces of the control, marine saline, and 5-HT groups (*n =* 5). (**C**) The dry weight of the 48-h accumulated feces of the control, DMSO + marine saline, and HC-030031 + 5-HT groups (*n =* 5). The symbols ** and *** mean *p <* 0.01 and *p <* 0.001, respectively (mean ± s.e.m.).

The HC-030031 group exhibited significantly lower dry feces weight than other groups (*df*1 = 2, *df*2 = 12, *F* = 9.571, both *p <* 0.01, Figure 6A). No significant difference was found between the control and DMSO groups (*p >* 0.05).

The dry weight of the 48-h accumulated feces was significantly higher in the 5-HT group than those in the control and marine saline groups (*df*1 = 2, *df*2 = 12, *F* = 14.773, both *p <* 0.001, Figure 6B). The dry feces weight showed no significance between the control and marine saline groups (*p >* 0.05).

There was no significant difference of the 48-h accumulated dry feces weight among control, DMSO + marine saline, and HC-030031 + 5-HT groups (*df*1 = 2, *df*2 = 12, *F* = 0.044, *p >* 0.05, Figure 6C).

These results show that gut emptying is positively correlated with TRPA1 and concentration of 5-HT in the gut of *S. intermedius*.

## 4. Discussion

Sea urchins are considered as “marine shredders” [2]. Their poor digestion and absorption of kelps make this energy source more available to a suite of other benthic detritivores, thus playing a positive role in the marine food webs. To understand the underlying molecular regulatory relationships behind this physiological characteristic, we investigated whether digestive enzyme activities and gut emptying are correlated with the reciprocal regulation of TRPA1 ion channels and 5-HT in the gut of *S. intermedius*.

### 4.1. TRPA1 Is Involved in the Digestion of S. intermedius

The food ingested by sea urchins is enclosed in a mucous membrane in the pharynx to form small pellets, which move through the pharynx and oesophagus to the intestinal tract where digestion and nutrient uptake occur [39]. Interestingly, the digestive enzyme activities (both pepsin and amylase) of *S. intermedius* with the highest expression of TRPA1 was significantly lower than that of *S. intermedius* with the lowest TRPA1 expression in the present study. This indicates that TRPA1 is involved in the food digestion and absorption of *S. intermedius*. This conclusion is not consistent with the finding of Fothergill et al. [40] that the TRPA1 channel in the digestive tract is activated by several compounds of mammal feed, thus improving their nutritional digestion efficiency. This disagreement suggests that the role of TRPA1 in digestive physiology is not conserved in the animal kingdom. The present finding suggests that TRPA1 expression varies among individuals under the same natural conditions. Considering that TRPA1 expression in the tube feet of *S. intermedius* shows a significant response to long-term low water temperature [27], we speculate that the expression pattern of TRPA1 in the gut tissues of *S. intermedius* is similar to that in tube feet. Individuals with higher TRPA1 expression are more sensitive to cold water temperature, which results in lower digestive enzyme activities and consequently play a negative role in the digestive function of sea urchins. It suggests that sea urchins probably have relatively poor absorption efficiency under the condition of cold water, producing more nutritious feces. This process provides more nutrients to a suite of benthic detritivores [13,41], playing a positive role in the marine food webs.

### 4.2. An Inter-Regulatory Relationship Exists between TRPA1 and 5-HT in the Gut of S. intermedius

The 5-HT level of *S. intermedius* with the highest expression of *TRPA1* was significantly higher than those with the lowest *TRPA1* expression. This indicates a link between *TRPA1* and 5-HT in the gut of *S. intermedius*. To further investigate whether an inter-regulatory mechanism exists in the gut of *S. intermedius*, we measured the *TRPA1* expression and 5-HT level in the gut of *S. intermedius* after the 5-HT and HC-030031 injection, respectively. As expected, the 5-HT level of sea urchins with TRPA1 inhibition was significantly lower than the control level, and the sea urchins with the 5-HT injection showed significantly higher *TRPA1* expression than the control level. This is consistent with the finding in rats (*Rattus norvegicus*) and mice (*Mus musculus*) that stimulation of TRPA1 evokes the release of 5-HT from cells and that TRPA1 in the gut was largely inhibited by the 5-HT receptor antagonists [25,31]. All these findings support the idea that an inter-regulatory mechanism exists between TRPA1 and 5-HT in the gut of *S. intermedius*, which offers an entry point to further investigate the molecular details of digestive physiology of sea urchins. This is important for food web stability in marine ecosystems.

### 4.3. Digestive Enzyme Activities Are Negatively Correlated with the TRPA1 and Concentration of 5-HT in the Gut of S. intermedius

The absorption and utilization of nutrients by *S. intermedius* are directly affected by digestive enzymes activities [15,16]. In this experiment, we used pepsin and amylase enzymes activities as two representative indicators to evaluate digestive and absorptive capacities of sea urchins. The inhibition of TRPA1 significantly contributes to higher digestive enzymes activities (pepsin and amylase) in the gut of *S. intermedius*. The phenomenon implies that digestive enzyme activities are negatively correlated with the TRPA1 expression in the gut of *S. intermedius*. However, the high expression of TRPA1 in cells of mammals (human, rat and mouse) increases gastric vagal nerve activity (GVNA), and promotes nutrient digestion and absorption [42,43]. This difference indicates that TRPA1 is probably not conservative in regulating digestion in various animals.

We subsequently investigated the relationship between 5-HT and digestive enzymes in the gut of *S. intermedius.* Sea urchins injected with 5-HT showed significantly lower activities of digestive enzymes. This suggests that 5-HT is involved in the digestive and absorptive capacities of *S. intermedius*. Similarly, 5-HT also play an important role in the food digestion and absorption of other animals. For example, dyspepsia was associated with the increasing of 5-HT in humans [44]. These findings suggest that the food digestion and absorption of *S. intermedius* was negatively correlated with 5-HT concentration in the gut.

To investigate whether a correlation exists between digestive enzymes activities and the reciprocal regulation of TRPA1 and 5-HT in the gut of *S. intermedius*, the HC-030031 + 5-HT experiment was carried out. We found that the positive effects of TRPA1 antagonist on the digestive enzyme activities were abolished after subsequent 5-HT injection. Taken together, digestive enzyme activities are negatively correlated with the TRPA1 and concentration of 5-HT in the gut of *S. intermedius*, thus negatively affecting their digestion and absorption.

### 4.4. Gut Emptying Is Positively Correlated with the TRPA1 and Concentration of 5-HT in the Gut of S. intermedius

In addition to digestive enzyme activities, gut emptying is another important indicator for food digestion and absorption of *S. intermedius.* This is supported by the finding that Antarctic sea urchins have a low-energy diet and require long periods of digestion [7]. This suggests that decreased gastric emptying is beneficial because it improves the absorption and utilization in sea urchins by increasing the time that food stays in the digestive tract [7]. In the present study, TRPA1 inhibition of *S. intermedius* produced significantly less feces (dry weight) within 48 h, indicating that a positive link exists between gut emptying and TRPA1 in the gut of the *S. intermedius*. Combined with the findings of this study regarding the role of TRPA1 in digestive enzyme activities, we conclude that the food digestion and absorption of *S. intermedius* are negatively correlated with TRPA1 expression in the gut. However, high expression of TRPA1 in digestive tract delayed gastric emptying in mice [45], which is contrary to our conclusion. The disagreement indicates that the role of TRPA1 in digestion and absorption is not conservative among animals, which supports the hypothesis that the role of TRPA1 in gastrointestinal motility depends on species [46]. *Strongylocentrotus intermedius* with 5-HT injection produced significantly more feces (dry weight) within 48 h, which is consistent with the result of 5-HT increased gastric emptying in rats [47]. This phenomenon is consistent with the finding that the 5-HT injection reduced the retention time of food in the gut of the Chinese mitten crab *Eriocheir sinensis* [29] and humans [48,49].

Further, we investigated whether a correlation exists between gut emptying and the reciprocal regulation of TRPA1 and 5-HT. As expected, the effect of TRPA1 antagonist on the gut emptying was abolished in *S. intermedius* treated with 5-HT. This result clearly indicates that gut emptying is positively correlated with TRPA1 and concentration of 5-HT in the gut of *S. intermedius*. It is also consistent with the finding that digestive enzyme activities are negatively correlated with the TRPA1 and concentration of 5-HT in the gut of *S. intermedius*, thus negatively impacting their digestion and absorption. On the contrary, TRPA1 delays gastric emptying in rats through 5-HT pathways [31]. This indicates that the relationship between gastrointestinal tract emptying and TRPA1 and 5-HT is species dependent.

Based on the results above, we conclude that the digestive enzyme activities and gut emptying are correlated with the reciprocal regulation of TRPA1 and 5-HT in the gut of *S. intermedius.* We speculate that if the molecules (TRPA1 and 5-HT) related to digestive physiology of sea urchins are affected, the digestive and absorptive functions of sea urchins are consequently altered. For example, *TRPA1* is highly expressed in the gut of sea urchins under cold water conditions, resulting in low digestive enzyme activities and fast gut emptying rates. This situation is unhelpful to the digestion and absorption of food nutrients by sea urchins, which reflects poor digestion and absorption capacities of sea urchins. This explains the observation that the food digestion and absorption of sea urchins are worse at low water temperatures than those at high water temperatures [50,51,52]. Ingestion rates and absorption efficiencies were lower in *L. variegatus* kept at the lower temperatures than those in *L. variegatus* kept at the higher temperatures [50]. Consistently, when *S. franciscanus* was fed with either a prepared diet or kelp *(N. luetkeana*), the feeding rates increased with increased water temperature [51]. Furthermore, there was a significant and linear increase in feed intake of *S. droebachiensis* with increased temperature, both in summer and winter [52]. Collectively, it suggests that the gut of sea urchins with high *TRPA1* expression probably have poorer digestion and absorption capacities under the low water temperature, producing feces with more energy and nutrients, thus providing relatively high-quality food for detritivores [14]. This bonus is critical to the benthic community, contributing to maintaining the stability of food webs in marine ecosystem.

## 5. Conclusions

The present study revealed three findings as follows: an inter-regulatory relationship exists between TRPA1 and 5-HT in the gut of *S. intermedius*, digestive enzyme activities are negatively correlated with the TRPA1 and concentration of 5-HT in the gut of *S. intermedius,* and gut emptying rate is positively correlated with TRPA1 and concentration of 5-HT in the gut of *S. intermedius*. Digestion and absorption of food are correlated with the reciprocal regulation of TRPA1 and 5-HT in the gut of *S. intermedius* and provide a baseline to further investigate the molecular details of digestive physiology of sea urchins. Furthermore, this novel finding explains the low food digestibility by sea urchins and provides valuable information to the digestive physiology of sea urchins. These results are key to understand the stability of food webs in the marine ecosystem.

## Figures and Tables

**Table 1:** PCR primers used in this study [27].

Primers	Sequences (5′-3′)	Application
TRPA1-F	GCCACCGCAGTCGTGTGTG	qPCR
TRPA1-R	TGGGCGTGGTCCGATAGTTAGTCTC	qPCR
18S rRNA-F	GTTCGAAGGCGATCAGATAC	Reference gene
18S rRNA-R	CTGTCAATCCTCACTGTGTC	Reference gene

## Data Availability

The datasets of the current study are available from the corresponding author on reasonable request.
